# Midlife perceived stress is associated with cognitive decline across three decades

**DOI:** 10.1186/s12877-023-03848-8

**Published:** 2023-03-04

**Authors:** Dinne Skjærlund Christensen, Ellen Garde, Hartwig Roman Siebner, Erik Lykke Mortensen

**Affiliations:** 1 Department of Psychology and Behavioural Science, Unit for Psychooncology and Health Psychology (Epos), Aarhus University, Bartholins Alle 11, Bld. 1351, 8000 Aarhus, Denmark; 2 Department of Oncology, Aarhus University Hospital, Palle Juul-Jensens Blvd. 99, 8200 Aarhus, Denmark; 3 Department of Public Health, Section of Environmental Health, University of Copenhagen, Øster Farimagsgade 5A, 1353 Copenhagen, Denmark; 4 Center for Healthy Aging, University of Copenhagen, Blegdamsvej 3B, 2700 Copenhagen, Denmark; 5 Danish Research Centre for Magnetic Resonance, Centre for Functional and Diagnostic Imaging and Research, Copenhagen University Hospital - Amager and Hvidovre, Kettegård Allé 30, 2650 Hvidovre, Denmark; 6 Department of Neurology, Copenhagen University Hospital Bispebjerg and Frederiksberg, Bispebjerg Bakke 23, 2400 Copenhagen, Denmark; 7 Department of Clinical Medicine, Faculty of Health and Medical Sciences, University of Copenhagen, Blegdamsvej 3B, 2200 Copenhagen N, Denmark

**Keywords:** Cognitive decline, Cognitive ability, Intelligence, Stress, Cohort study

## Abstract

**Background:**

Research indicates detrimental effects of stress on brain health and cognitive functioning, but population-based studies using comprehensive measures of cognitive decline is lacking. The present study examined the association of midlife perceived stress with cognitive decline from young adulthood to late midlife, controlling for early life circumstances, education and trait stress (neuroticism).

**Methods:**

The sample consisted of 292 members of the Copenhagen Perinatal Cohort (1959–1961) with continued participation in two subsequent follow-up studies. Cognitive ability was assessed in young adulthood (mean age 27 years) and midlife (mean age 56 years) using the full Wechsler Adult Intelligence Scale (WAIS), and perceived stress was measured at midlife using the Perceived Stress Scale. The association of midlife perceived stress with decline in Verbal, Performance and Full-Scale IQ was assessed in multiple regression models based on Full Information Maximum Likelihood estimation.

**Results:**

Over a mean retest interval of 29 years, average decline in IQ score was 2.42 (SD 7.98) in Verbal IQ and 8.87 (SD 9.37) in Performance IQ. Mean decline in Full-scale IQ was 5.63 (SD 7.48), with a retest correlation of 0.83. Controlling for parental socio-economic position, education and young adult IQ, higher perceived stress at midlife was significantly associated with greater decline in Verbal (β = − 0.012), Performance (β = − 0.025), and Full-scale IQ (β = − 0.021), all *p* < .05. Across IQ scales, additionally controlling for neuroticism in young adulthood and change in neuroticism had only minor effects on the association of midlife perceived stress with decline.

**Conclusions:**

Despite very high retest correlations, decline was observed on all WAIS IQ scales. In fully adjusted models, higher midlife perceived stress was associated with greater decline on all scales, indicating a negative association of stress with cognitive ability. The association was strongest for Performance and Full-scale IQ, perhaps reflecting the greater decline on these IQ scales compared to Verbal IQ.

**Supplementary Information:**

The online version contains supplementary material available at 10.1186/s12877-023-03848-8.

With an increasingly aging population globally [1], more individuals will be affected by cognitive decline, and the prediction and early-stage prevention of cognitive decline thus becomes of central importance. Long-term stress represents a potential risk factor for cognitive decline as it may have adverse effects on brain health and cognitive functioning via two mechanisms; indirectly via other stress-related disorders and behaviors [2, 3] and directly via stress-related effects on the brain. Chronic psychological stress elicits sustained activation of the hypothalamic–pituitary–adrenal (HPA) axis, with adrenal release of glucocorticoids such as cortisol. In line with the glucocorticoid-cascade hypothesis [4], numerous studies in both animals and humans have shown that prolonged cortisol elevations are associated with hippocampal atrophy and memory impairments [5–7], and cognitive decline [8, 9]. Adult individuals who suffer from stress-related exhaustion [10], chronic stress [11], or posttraumatic stress disorder (PTSD) [12], show poorer performance in some cognitive tests compared to healthy controls. Further, there is a consistent link between chronic stress and pathological brain aging, and stress is a well-established predictor of dementia [13–15]. However, research in humans has mainly focused on the influence of early life stress on later life cognitive function and performance [7]. Thus, in order to identify effective intervention strategies, there is a need for studies on effects of exposures to potential stress at later stages, focusing on the subtle decline occurring already at midlife.

Research into the associations of stress with more comprehensive measures of cognitive ability and decline is also lacking. Most studies on stress and cognition are based on limited measures of cognitive function acquired at a single time point, thus not examining decline [16–18]. The few longitudinal studies assessed changes in cognition over relatively short periods [19, 20]. This is problematic because low IQ predicts increased stress exposure, e.g. in the form of traumatic events [21]. Thus, early intelligence may confound concurrent associations of stress with cognitive ability, and needs to be taken into consideration when examining cognitive dysfunction as a symptom or outcome of stress during adulthood.

Several studies did not include measures of stable personality characteristics, although certain personality traits are associated with both stress and, to a lesser extent, cognitive ability [19, 20, 22]. Trait neuroticism, in particular, is closely associated with stress, as it is partly characterized by the tendency to experience psychological stress [23]. Neuroticism also tends to be inversely associated with cognitive ability [24–26], and the aspects of neuroticism associated with proneness to distress have been found to be associated with increased cognitive decline [27]. Similarly, early life events and circumstances may also confound the association of stress with cognitive function, as adverse childhood circumstances, such as poverty, are associated both with poorer cognitive function [28, 29] and perceived stress later in life [30]. Finally, compared to studies on patient groups, there are fewer studies based on the general population. As a result, the extent to which subclinical levels of psychological stress influence cognitive function thus remains relatively unexplored.

Using a longitudinal study design that was not affected by the limitations mentioned above, Aggarwal et al. [31] examined perceived stress as a predictor of cognitive decline in a population-based study. Higher levels of perceived stress were found to be associated with lower initial cognitive scores and accelerated cognitive decline over 7 years. However, the study was hampered by other limitations. Firstly, the measures of perceived stress and cognitive function were somewhat limited. A 6-item version of the 10-item Perceived Stress Scale (PSS) was used [32, 33], and cognitive function was assessed by four brief tests, including a test of perceptual speed, two tests of episodic memory, and the Mini Mental State Examination (MMSE) [34], a screening tool for cognitive impairment. The assessment thus left several aspects of both perceived stress and cognitive functioning uncovered. Secondly, the study was based on a population aged 65 years or older, with a mean age of 72 years. While this is highly relevant for the study of age-related cognitive decline, it does not add to our understanding of the association of stress with decline from peak performance in young adulthood over a substantial part of the lifespan to late middle-age [35]. Furthermore, in a population with a mean age of 72 years, many individuals will present cognitive deficits already at the baseline cognitive assessment. As such deficits may themselves induce stress, they substantially increase the risk of reverse causation, as demonstrated by a recent study showing PSS scores to be significantly higher in individuals with subjective cognitive decline [36]. Finally, while the study did take several potential confounders into account, including neuroticism (though again using a limited, four-item measure) and health history, no measure of early life factors was controlled for, despite such factors being arguably more likely to confound the stress-cognitive function association than e.g., smoking, which was controlled for. Specifically, measures of childhood socioeconomic position have been found to be significantly associated with cognitive functioning in middle age [29] and perceived stress in late adulthood [37].

In the present study, we examined the association of perceived stress at midlife with cognitive decline from young adulthood to late midlife using a comprehensive measure of cognitive ability. To overcome the limitations of the previously published study by Aggarwal et al. [31], we assessed midlife perceived stress with the full PSS and cognitive ability with the full Wechsler Adult Intelligence Scale (WAIS), and controlled for early life circumstances. We hypothesized higher levels of stress to be associated with greater decline in Verbal, Performance and Full-scale IQ. Further, we examined the influence of trait stress (neuroticism) on this association, both prospectively and cross-sectionally.

## Methods

### Sample

The sample consisted of 292 members of the Copenhagen Perinatal Cohort (CPC, 1959–1961) with continued participation in two subsequent follow-up studies: the Prenatal Development Project (PDP, 1982–1994) and the Early Life Determinants of Midlife Mental Development and Brain Structure study (LifeMabs, 2015–2018).

The CPC consists of 9125 children born at the Copenhagen University Hospital during the period October 1959 to December 1961 [38]. It contains information on pre-, peri- and postnatal factors collected during pregnancy, at the time of birth and at a 1-year follow-up. The PDP was designed to examine the long-term effects of early life factors on psychological and physical development [39]. A total of 1575 members of the CPC were invited to the PDP, and a total of 1208 members agreed to participate. Cognitive ability was assessed for 1155 of these participants. LifeMabs was established in 2015 as a follow-up of the PDP. A total of 1024 PDP-members were invited to participate (the remaining had either died or emigrated), 368 of whom completed the full assessment or part of the study. For 76 of the participants, data were incomplete on stress or cognitive ability, leaving a final sample of 292 participants.

LifeMabs was approved by the Danish National Committee on Health Research Ethics (No: H-15012317) and Danish Data Protection Agency (No: 2013-41-2593) and all participants signed informed consent forms.

### Measures

#### Cognitive ability

Both young adult and midlife cognitive ability were assessed using the original WAIS [40]. The test consists of six verbal subtests (Information, Comprehension, Similarities, Arithmetic, Digit Span, Vocabulary) and five performance subtests (Digit-symbol, Picture Completion, Block Design, Picture Arrangement, and Object Assembly). From these subtests, Verbal, Performance and Full-scale IQ scores were calculated. Danish test score norms were developed as part of the PDP and based on an education representative sample of 400 men and women between the ages of 20 to 34 years. To allow comparison between the follow-ups, IQ scores from both young adulthood (PDP assessment) and midlife (LifeMabs assessment) were based on these norms. The WAIS was individually administered by three psychologists in PDP and by either a psychologist or three psychology students in LifeMabs.

#### Midlife perceived stress

Midlife perceived stress was measured as part of the LifeMabs study using the Danish version of Cohen’s PSS [32, 33]. It consists of 10 items with 5-point Likert scale responses (from 0 = “never”, to 4 = “very often”), yielding a full scale range of 0–40. Higher scores indicate greater levels of perceived stress. The PSS can be used to assess the degree to which the respondent experiences life to be stressful, that is, unpredictable, uncontrollable and overloading [32], over the past 4 weeks. The Danish version has been validated [41]. The PSS was completed by the participants as part of an online questionnaire immediately after the WAIS test administration.

#### Covariates

Information on parental socioeconomic position (SEP) in infancy was used as an indicator of early life circumstances. This was available from the CPC 1-year follow-up, and was based on four factors: occupation of the breadwinner, type of income of the breadwinner (e.g. unemployment relief, weekly or monthly wage, own business or capital etc.), education of the breadwinner and quality of living accommodation (size, number of persons per room etc.). Each factor was scored from 0 to 5, and the resulting 0–20 point scale was recoded to a scale from 1 to 8, with higher values reflecting higher levels of SEP [38]. Because it is difficult to separate exposure to stress over the life course and current level of stress, we included a measure of trait stress in the analyses. This measure was neuroticism assessed by a Danish version of the Eysenck Personality Questionnaire (EPQ) [42] both in the PDP and the LifeMabs study. The neuroticism scale consists of 23 yes/no items. Coefficient alpha was .87 for the PDP assessment and .89 for the LifeMabs assessment. Additional covariates were sex, age at young adult assessment, retest interval, and education.

### Data analysis

Independent samples t-tests were used to compare LifeMabs participants with complete and incomplete data and to compare LifeMabs participants and non-participants. Pearson bivariate correlations were used to analyze associations among the PSS, the WAIS IQs, and the included covariates. Analyses of WAIS IQ changes were conducted using change scores [43, 44]. For each respective intelligence scale (Verbal, Performance and Full-scale), a change score was calculated by subtracting the young adulthood score from the midlife score. For each of these change scores, a series of three nested multiple regression models were analyzed: Model 1 regressed the observed change score on sex, age at the time of young adult assessment, retest interval, parental SEP, education and midlife perceived stress (PSS). Model 2 additionally included young adult IQ (Verbal, Performance or Full-scale for the three change scores respectively). In model 3, trait stress was adjusted for by further including young adult neuroticism and change in neuroticism from young adulthood to midlife. Interactions between midlife perceived stress and sex, as well as between midlife perceived stress and young adult IQ scores were tested in model 2 and were not significant.

Though the sample was restricted to participants with available data on cognitive ability and midlife perceived stress, there were missing data on some of the covariates. While the missing data rate was generally below 1, 13% had missing data on parental SEP. For this reason, all models were based on full information maximum likelihood (FIML), which enables the inclusion of incomplete data under the missing at random assumption [45].

To a get a more detailed picture of associations of perceived stress with cognitive function, models 1–3 were also analyzed with decline in each of the 11 WAIS subtests as outcome in supplementary analyses (see Table S1). All analyses were conducted in Stata V17 (StataCorp. 2015, Texas, USA).

## Results

Independent samples t-tests of the variables included in Table 1 and the WAIS IQs showed no significant differences between LifeMabs participants with complete data and participants without complete data. However, analyses comparing young adult data for LifeMabs participants and non-participants showed significantly higher Parental SEP and IQ among participants, corresponding to a 5-6 IQ point difference in mean IQ on all WAIS IQs (sex, test age, and neuroticism score did not differ significantly between participants and non-participants).

**Table 1:** Descriptive statistics

Variable	N	Mean (SD) or N (%)	Correlation with young adult Verbal IQ	Correlation with midlife Verbal IQ	Correlations with young adult Performance IQ	Correlation with midlife Performance IQ
Sex (F = 1/M = 0)	292	146 (50%)	−0.22***	− 0.19**	− 0.05	−0.02
Midlife test age	292	56.45 (0.76)	0.20***	0.18**	0.14*	0.13*
Young adult test age	292	27.34 (4.24)	0.07	0.04	0.00	−0.01
Retest interval, years	292	29.11 (4.05)	−0.03	− 0.00	0.02	0.04
Midlife perceived stress	292	10.19 (6.98)	−0.29***	− 0.28***	− 0.15**	− 0.26***
Young adult neuroticism	292	7.37 (4.87)	−0.31***	− 0.25***	− 0.09	− 0.11
Midlife neuroticism	290	5.67 (5.05)	− 0.23***	−0.15**	− 0.07	−0.16**
Change in neuroticism	290	−1.68 (4.52)	0.03	0.01	0.02	−0.08
Education	291	11.12 (1.59)	0.57***	0.56***	0.37***	0.35***
Parental SEP	253	4.83 (1.85)	0.39***	0.46***	0.16*	0.20**

*SEP* Socioeconomic position

*p < 0.05, **p < 0.01, ***p < 0.001

Sample characteristics and correlations with young adult and midlife Verbal and Performance IQ are displayed in Table 1. The sample was evenly distributed in terms of sex while the mean age at young adult follow-up was 27.34 years and at the midlife assessment 56.45 years, resulting in a mean retest interval of 29.11 years. The mean midlife PSS score was 10.19, which is slightly lower than the reported means of three large probability-based American samples [46] and equivalent to that of a national representative Danish sample [47]. There was a decline in mean EPQ neuroticism from 7.37 in young adulthood to 5.67 in midlife. Midlife PSS score correlated negatively with both young adult and midlife Verbal and Performance IQs, and the two measures of neuroticism correlated negatively with both young adult and midlife verbal IQs. For Performance IQ, only midlife neuroticism correlated significantly with midlife Performance IQ. Midlife test age, years of education and parental SEP showed significant positive correlations with young adult and midlife IQs.

Table 2 presents the young adult and midlife mean scores for all IQ scales. The young adult mean IQs were about one third of the theoretical standard deviation of 15 above 100, indicating that the participants were well-functioning as young adults. All IQ scales showed significant decline, but there were substantial differences between the scales. Thus, the mean decline scores were 2.42, 8.87, and 5.63 for the Verbal, Performance and Full-scale IQs, corresponding to 0.16, 0.59 and 0.38 of a theoretical standard deviation of 15. In spite of the observed decline, Table 2 shows high retest correlations, reflecting rank order stability of the WAIS IQs over an average retest interval of 29 years, possibly diluted by individual differences in age-related cognitive decline. Table 2 also shows significant negative correlations between young adult IQ scores and the corresponding decline scores.

**Table 2:** WAIS retest data for 292 participants

	Young adult IQ Mean (SD)	Midlife IQ Mean (SD)	Difference Mean (SD)	Retest r (95% CI)	Young adult IQ x Difference r
Verbal IQ	105.84 (13.47)	103.41 (12.71)	2.42 (7.98)***	0.82 (0.77;0.85)	−0.39 (− 0.48;-0.29)
Performance IQ	105.71 (12.43)	96.83 (13.74)	8.87 (9.37)***	0.75 (0.69;0.80)	−0.23 (− 0.34;-0.12)
Full-scale IQ	106.33 (12.65)	100.71 (12.64)	5.63 (7.48)***	0.83 (0.79;0.86)	−0.30 (− 0.40;-0.19)

*WAIS* Wechsler Adult Intelligence Scale

*** indicates *p* < 0.001

Table 3 shows the pairwise correlations among study variables. Despite the significant correlations of midlife PSS and several covariates with young adult IQs shown in Table 1, there were no significant correlations with Full-scale IQ change scores. However, both midlife PSS and midlife neuroticism score showed significant negative correlations with Performance IQ change scores (*r* = −.17 and − .14, respectively). As expected, midlife PSS was significantly associated with both young adult and midlife neuroticism score (*r* = .46 and .60, respectively).

**Table 3:** Pairwise correlations

	1	2	3	4	5	6	7	8	9	10	11	12	13
1. Full-scale IQ change	1.00												
2. Verbal IQ scale change	0.82***	1.00											
3. Performance IQ scale change	0.76***	0.25***	1.00										
4. Sex (F = 1/M = 0)	0.08	0.07	0.05	1.00									
5. Midlife test age	−0.03	−0.05	−0.00	−0.17**	1.00								
6. Young adult test age	−0.05	−0.06	−0.02	−0.14*	0.33***	1.00							
7. Retest interval	0.04	0.05	0.02	0.11	−0.16**	−0.98***	1.00						
8. Midlife perceived stress	−0.07	0.04	−0.17**	0.26***	−0.02	−0.10	0.10	1.00					
9. Young adult neuroticism	0.06	0.13*	0.04	0.35***	−0.10	−0.25***	0.24***	0.46***	1.00				
10. Midlife neuroticism	0.02	0.15*	−0.14*	0.35***	−0.01	−0.05	0.05	0.60***	0.57***	1.00			
11. Change in neuroticism	−0.10	−0.04	−0.14*	−0.10	0.05	0.14*	−0.13*	0.15*	−0.42***	0.26***	1.00		
12. Education	−0.05	−0.08	0.01	0.03	0.02	−0.07	0.08	−0.09	−0.12*	−0.05	0.03	1.00	
13. Parental SEP	0.09	0.06	0.08	−0.00	0.06	−0.12*	0.14*	0.03	−0.06	0.00	−0.00	0.49***	1.00

*p < 0.05, **p < 0.01, ***p < 0.001

Table 4a shows the results of models 1–3 for Verbal IQ score change. Standardized coefficients are shown. Midlife PSS was only significantly associated with change in Verbal IQ when young adult baseline Verbal IQ was included as covariate in model 2 and 3. Higher levels of midlife stress predicted greater levels of decline, but midlife perceived stress only explained 1.4% of the variance in the fully adjusted model. The association was slightly strengthened when adjusting for neuroticism in young adulthood and change in neuroticism from young adulthood to midlife, though neither of these significantly predicted Verbal IQ change. For Verbal IQ change, the only other significant predictors were parental SEP and education. Both of these predicted less decline in models 2 and 3, and education predicted more decline in the model without young adult Verbal IQ as covariate.

**Table 4:** Verbal, Performance and Full-scale IQ change scores regressed on perceived stress and covariates

	Model 1		Model 2		Model 3	
**a**) **Verbal IQ change**	β	*95% CI*	β	*95% CI*	β	*95% CI*
Sex (F = 1/M = 0)	0.07	−0.05;0.19	−0.03	−0.13;0.08	−0.04	−0.15;0.07
Young adult test age	−0.03	−0.15;0.09	−0.00	−0.11:0.10	0.01	−0.10;0.12
Midlife test age	−0.03	−0.16;0.09	0.05	−0.06;0.16	0.05	−0.06;0.16
Parental SEP	0.15*	0.01;0.29	0.24^***^	0.12;0.37	0.25^***^	0.12;0.37
Education	−0.16*	−0.29;-0.02	0.15*	0.02;0.29	0.15*	0.02;0.29
Midlife perceived stress (PSS)	0.00	−0.12;0.12	−0.12*	−0.23;-0.01	−0.15*	−0.27;-0.02
Young adult verbal IQ			−0.62***	−0.75;-0.50	−0.61***	−0.73;-0.49
Young adult neuroticism					0.06	−0.09:0.21
Neuroticism change					0.01	−0.12;0.13
*Explained variance (R*^*2*^*)*	*3.2%*		*24.0%*		*24.3%*	
*Explained by PSS*	*0.0%*		*1.3%*		*1.4%*	
**b) Performance IQ change**						
Sex (F = 1/M = 0)	0.10	−0.02;0.22	0.10	−0.02;0.21	0.09	−0.02;0.21
Young adult test age	−0.02	−0.14;0.10	−0.03	−0.15;0.08	−0.02	−0.14;0.09
Midlife test age	0.01	−0.11;0.13	0.06	−0.06;0.17	0.05	−0.06;0.17
Parental SEP	0.12	−0.02;0.26	0.11	−0.03;0.24	0.11	−0.03;0.24
Education	−0.07	−0.21;0.06	0.04	−0.09;0.18	0.04	−0.09;0.18
Midlife perceived stress (PSS)	−0.21***	−0.33;-0.10	−0.25***	−0.35;-0.13	−0.20**	−0.34;-0.06
Young adult performance IQ			−0.30***	−0.42;-0.19	−0.30***	−0.41;-0.18
Young adul neuroticism					−0.05	−0.20;0.11
Neuroticism change					−0.12	−0.26;0.01
*Explained variance (R*^*2*^*)*	*5.0%*		*12.5%*		*13.7%*	
*Explained by PSS*	*4.0%*		*5.4%*		*2.6%*	
	Model 1		Model 2		Model 3	
**c) Full-scale IQ change**	β	*95% CI*	β	*95% CI*	β	*95% CI*
Sex (F = 1/M = 0)	0.11	−0.01;0.22	0.05	−0.06;0.17	0.04	−0.07;0.15
Young adult test age	−0.03	−0.15;0.09	−0.02	−0.14;0.09	−0.01	−0.12;0.11
Midlife test age	−0.01	−0.13;0.11	−0.06	−0.05;0.18	0.06	−0.05;0.17
Parental SEP	0.17*	0.03;0.30	0.21**	0.08;0.34	0.22**	0.09;0.34
Education	−0.15*	−0.28;-0.02	0.10	−0.04;0.24	0.10	−0.04;0.24
Midlife perceived stress (PSS)	−0.12*	−0.24;-0.00	−0.21***	−0.32;-0.10	−0.21**	−0.34;-0.08
Young adult Full-scale IQ			−0.48***	−0.61;-0.35	−0.47***	−0.60;-0.35
Young adult neuroticism					0.03	−0.12;0.19
Neuroticism change					−0.05	−0.18;0.08
*Explained variance (R*^*2*^*)*	*4.1%*		*17.5%*		*18.2%*	
*Explained by PSS*	*1.3%*		*3.8%*		*3.0%*	

*SEP* Socioeconomic position. Standardized beta coefficients

*p < 0.05, **p < 0.01, ***p < 0.001

Table 4b presents the results for changes in Performance IQ. Except for young adult Performance IQ, midlife PSS was the only significant predictor, predicting more decline in all models and explaining 2.6 to 5.4% of the variance in Performance IQ change scores. The association became slightly weaker when adjusting for neuroticism in young adulthood and change in neuroticism.

As shown in table 4c, the results for Full-scale IQ change score were very similar to those for the two IQ subscales. Midlife PSS was significantly associated with more decline in all models and explained between 1.3 and 3.8% of the variance in Full-scale IQ change scores. Again, the association of midlife PSS with Full-scale IQ change score remained significant when adjusting for neuroticism and change in neuroticism.

As a sensitivity analysis, a model without adjustment for young adult IQ, but with adjustment for neuroticism and change in neuroticism was analyzed for all IQs scales. For this model, midlife PSS was significantly associated with change in Performance IQ and marginally significant for the Full Scale IQ (results not shown). Results of supplementary analyses provided in Table S1 showed that regression coefficients for all individual WAIS subtests indicated that higher midlife PSS scores were associated with more cognitive decline in models adjusting for young adult IQ. However, the associations were only significant for two verbal subtests (Arithmetic and Digit Span), but significant for all performance subtests except Block Design.

Results from complete case analyses were highly similar to those based on FIML estimation. These results are available from the first author (DSC) by demand.

## Discussion

In this study on stress and cognitive decline, a significant decline in intelligence from young adulthood to late midlife was observed on all WAIS IQ scales, but substantially more decline on Performance IQ than Verbal IQ. The study thus corroborates previous studies observing relatively early midlife decline in some cognitive functions [48]. However, the study also observed relatively high retest correlations over an average follow-up interval of 29 years, confirming the stability of individual differences in intelligence and suggesting that individual differences in cognitive decline are relatively modest. This may be one of the reasons that it has proved difficult to identify consistent predictors of age-related cognitive decline [49]. Indeed, only a few significant predictors were identified in the present study apart from parental SEP and education and the consistent association between higher young adult IQ and larger decline, confirming previous Danish findings [50].

On this background the consistent association between midlife perceived stress and larger decline merits attention. The association between midlife perceived stress and individual IQ became stronger in models adjusting for young adult IQ and remained significant when adjusted for trait stress in the form of neuroticism. Our findings are consistent with recent studies. Aggarwal et al. [31] showed that perceived stress was associated with decline in global cognitive function in a population aged 65 years and above. Munoz et al. [20] found that global perceived stress predicted cognitive slowing across 2 years in older adults (mean age 80 years). Likewise, a more recent study demonstrated that perceived stress predicted decline in executive functioning across a 6-year interval, also in an older population (mean age of 74 years at first wave) [22].

Our findings extend these previous findings by demonstrating an association between perceived stress and IQ in a younger sample with a substantially longer follow-up interval and a comprehensive measure of cognitive ability. While the association was significant for all three IQ measures, it was strongest for Performance and Full-scale IQ, perhaps reflecting the more substantial decline on these IQ scales. In apparent contrast to our findings, Chen et al. [19] found no support for their hypothesis that level of perceived stress would be associated with faster cognitive decline in episodic memory, perceptual speed, and working memory in older adults (mean age 73 years). As the authors themselves mention, this null finding might be attributed to the relatively short follow-up of just two years. Yet, a lack of association between perceived stress and decline in episodic memory, word fluency and block design test was also found in a 10-year longitudinal study of middle-aged swedes (40-60 years) [51].

Several factors may contribute to the inconsistent findings. The assessment of perceived stress varies between studies. In the present study, midlife stress was measured using the PSS. While this inventory is widely used and well-validated, it is a state measure, which is not designed to capture chronic stress. Although a pure state measure can only explain state-related cognitive decline, Munoz et al. [20] propose that higher PSS scores reflect both enduring environmental factors and stable individual differences which may exacerbate the stress response and accumulate over time [20]. In our study both young adult and midlife neuroticism correlated substantially with midlife perceived stress, suggesting that stable individual differences in appraisal and coping with stress contribute an enduring component to state measures of perceived stress. In fact, a recent study suggested that perceived stress mediates the association between neuroticism and cognitive decline [52], with higher neuroticism increasing perceived stress, thereby leading to poorer cognition. However, consistent with the findings by Aggarwal et al. [31], we did not find indication that the association of stress with cognitive change was substantially confounded by neuroticism, though the association of midlife perceived stress with Performance IQ change was slightly attenuated. This may reflect weak correlations between neuroticism and cognitive decline and is in line with the inconsistent evidence on the influence of neuroticism or trait stress on cognitive decline. Thus, a study by Wilson et al. [27] found that the aspects of neuroticism associated with proneness to distress were associated with increased cognitive decline, while a longitudinal study with 30 year follow-up observed no significant associations [53]. In our adjusted models, neither young adult neuroticism nor change in neuroticism was significantly associated with cognitive change, though associations of Verbal and Full-scale IQ with neuroticism were significant both in young adulthood and midlife.

It is worth noting that the PSS is a measure of *perceived* stress, and thus does not directly reflect objective stress exposures such as major life events. The effects of perceived stress may to some extent reflect effects on test performance of acute stress, but also stable individual differences in the experience of stress during everyday life, although the results were little affected by inclusion of neuroticism as a measure of trait stress. Previous findings regarding the association of more objective measures of stress with cognitive decline have been mixed, including both null-findings [54], positive associations (that is, higher levels of decline associated with more stress exposure) in either the full sample [55] or a cognitively impaired subsample [56], and differential effects of types of life events on decline [57]. One study found the effect of life events to be moderated by age and education, perhaps explaining the otherwise inconsistent findings [58]. Taken together, the literature on objective stress seems to indicate that exposure in itself does not predict cognitive decline, and it has been argued that the use of subjective appraisals of stress increases the predictive validity of stress measurements [55].

Assessment of cognitive decline also varies among studies. Several studies include one or more tests of episodic memory supplemented by two or three other tests assumed to be sensitive to cognitive aging [19, 31, 51, 59]. In a few studies global cognitive measures have been derived [31], but it is generally rare for studies to include comprehensive assessment of intelligence, although there is increasing evidence that age-related cognitive decline in different domains tend to be correlated [60]. This raises the issue of whether perceived stress is associated with global cognitive decline or decline in specific tests and cognitive functions. Our results are somewhat ambiguous since associations were significant for both Verbal and Performance IQ, but stronger for Performance IQ. Analyses of individual subtests confirmed these trends, which may reflect global effects that were only significant on the most age-sensitive subtests or effects limited to specific functions. To the extent the PSS measure of perceived stress primarily reflects acute stress, effects on tests requiring attention, concentration, and speed would be expected. This roughly corresponds to our pattern of results for the performance subtests as well as the significant associations for Arithmetic and Digit Span, and would imply that effects of perceived stress on cognitive decline are to some extent reversible. This possibility is corroborated by evidence in rodents and humans suggesting the effects of stress on the brain and cognitive performance to be reversible in the absence of stress, though perhaps less so for stress exposure in early life [7, 61]. We found parental SEP to be a consistent predictor of decline in Verbal IQ, possibility reflecting effects of early life exposure to adverse circumstances and stress.

In addition to the variance in the applied cognitive tests, studies also vary substantially with respect to age at baseline assessment and length of the retest interval. Higher initial age is often associated with relatively short retest intervals, which in some cases have only been 2 years [19, 20]. Obviously, studies of effects of perceived stress on decline over a two-year period in a 60-year or older sample is not comparable to studies of decline from young adulthood over a period of three decades. However, in older samples decline is expected to be more substantial over shorter periods, and the amount of decline may be an important factor influencing the power to detect associations with perceived stress. Indeed, one interpretation of the pattern in our results is that effects are global, but associations with perceived stress are stronger for the Performance IQ and subtests showing most decline.

### Strengths and limitations

This study contributes to the existing literature in several ways. The main limitations of previous studies were addressed by using comprehensive and extensively validated measures of stress, cognitive ability and neuroticism, as well as controlling for potential confounding by early life circumstances. Further, the study covers a substantial timespan from young adulthood to late midlife, evading practice effects while adding insight to the stress-decline association in a younger population than hitherto examined.

However, the study has several limitations which, in addition to the relatively small sample, complicates the interpretation of the findings. The included measure of perceived stress was measured only once, and reflects acute (rather than chronic) stress. Further, for practical reasons and because accuracy of the WAIS test was prioritized, PSS was measured immediately after the WAIS test. Thus, despite being asked about perceived stress over the past 4 weeks, potential stress experienced as part of the WAIS administration may have caused some participants to score higher on the PSS. In addition to the observational nature of the data, this means that we cannot establish causality, nor exclude that cognitive decline in fact influences midlife stress rather than vice versa, as the association is most likely bidirectional. Future work should be based on longitudinal data, preferably with multiple measurements of both stress and cognitive ability. Further, several probable mechanisms remain unexplored in the present study, including health-related behaviors such as sleep, smoking and (reduced) physical activity, changes in cognitively stimulating leisure activities, and chronic diseases, which may all influence cognitive decline or reflect indirect effects of stress on cognitive decline. Finally, generalizability of the present results may be influenced by selection bias. Especially in older populations, study participation is associated with higher cognitive abilities [62], perhaps causing the (otherwise curious) positive association between midlife test age and Full-scale IQ shown in Table 1. However, the limited midlife age range of 4 years makes this difficult to interpret, and it would not explain the association with young adult IQ of similar magnitude. Further, the standard deviations of the young adult and midlife IQ scores were highly similar (see Table 2), indicating that participation at midlife is not greatly influenced by selection related to IQ. Selection may also arise in relation to perceived stress; it is conceivable that persons with high levels of midlife perceived stress are less inclined to participate. If so, this would likely cause the association of midlife perceived stress with cognitive decline to be underestimated.

## Conclusion

This study observed significant decline from young adulthood to late midlife on all WAIS IQ scales, and decline was substantial on Performance IQ (0.59 standard deviation). Parental SEP was a consistent predictor of Verbal IQ while perceived stress at midlife was a consistent predictor of decline in both Verbal and Performance IQ.

Cognitive decline negatively impacts individual levels of daily functioning [63], quality of life, and incurs substantial costs to society [64]. Our findings suggest a negative association of midlife perceived stress with cognitive ability, underlining the importance of targeting stress in the general population.

## Supplementary Information

**Additional file 1: Table S1.** Midlife PSS coefficients for WAIS subtest change scores.

## Data Availability

The datasets generated and/or analyzed in the current study are not publicly available due to the sensitive nature of the data and in protection of the anonymity of the participants, but are available from the corresponding author on reasonable request.

## Abbreviations

CPC: Copenhagen Perinatal Cohort
EPQ: Eysenck Personality Questionnaire
FIML: Full Information Maximum Likelihood
HPA: Hypothalamic–pituitary–adrenal
LifeMabs: Early Life Determinants of Midlife Mental Development and Brain Structure study
MMSE: Mini Mental State Examination
PDP: Prenatal Development Project
PTSD: Posttraumatic stress disorder
PSS: Perceived Stress Scale
SEP: Socioeconomic position
WAIS: Wechsler Adult Intelligence Scale
