# Supplementary material for: Midlife perceived stress is associated with cognitive decline across three decades
Source: BMC Geriatr. 2023 Mar 4;23:121. doi: 10.1186/s12877-023-03848-8 (PMC9985854; doi:10.1186/s12877-023-03848-8)
Supplement: Supplementary file 1 — Additional file 1: Table S1. Midlife PSS coefficients for WAIS subtest change scores. [file 12877_2023_3848_MOESM1_ESM.docx]

| **Table S1:** Midlife PSS coefficients for WAIS subtest change scores | | | |
| --- | --- | --- | --- |
|  | Model 1^a^ | Model 2^b^ | Model 3^c^ |
|  | β | β | β |
| Information | 0.02 | -0.06 | -0.08 |
| Comprehension | -0.01 | -0.10 | -0.08 |
| Arithmetic | -0.00 | -0.14** | -0.11 |
| Similarities | 0.09 | -0.01 | -0.06 |
| Digit Span | -0.08 | 0.14* | 0.16* |
| Vocabulary | 0.01 | -0.06 | -0.04 |
| Digit Symbol | -0.12* | -0.17** | -0.16* |
| Incomplete Pictures | -0.10 | -0.15** | -0.08 |
| Block Design | -0.05 | -0.08 | -0.11 |
| Picture Arrangement | -0.11 | -0.15** | -0.20*** |
| Object Assembly | -0.13 | -0.13* | -0.08 |

*Note.* PSS = Perceived Stress Scale. ^a^Adjusted for sex, young adult age, midlife age, parental SEP, education and midlife perceived stress (PSS). ^b^Model 1 + young adult subtest score. ^c^Model 2 + young adult neuroticism and change in neuroticism from young adulthood to midlife. **p* < 0.05, ***p* < 0.01, ****p* < 0.001
